# Diet Quality and Mental Health Amongst Acute Inpatient Psychiatric Patients

**DOI:** 10.7759/cureus.12434

**Published:** 2021-01-02

**Authors:** Raveena Gill, Sean F Tyndall, Darshini Vora, Rashedul Hasan, James L Megna, Luba Leontieva

**Affiliations:** 1 Psychiatry, State University of New York Upstate Medical University, Syracuse, USA; 2 Psychiatry, State University of New York Upstate Medical University, Syracuse, USA

**Keywords:** inpatient psychiatry, mental health, dietary habits

## Abstract

This is the first study that explored the self-reported dietary habits of acute psychiatric inpatients. We found that 75% of the psychiatric inpatients have an unhealthy diet, which correlates with higher body mass index (BMI) and lower education level. We also found an increased sugar consumption in inpatients with schizophrenia.

The link between nutrition and mental health has been explored to a limited extent owing to the cumbersome nature of conducting research that involves specific dietary intervention and follow up. Yet, there is existing literature linking poor diet with impaired mental health and poor recovery from depression, amongst other disorders. Good nutrition can be exemplified with diets like the Mediterranean diet with a focus on certain food groups that provide the nutrients linked to neurotransmitters and a fairly new concept of the gut-brain axis.

A Mediterranean-style dietary intervention supplemented with fish oil improves diet quality and mental health in people with depression. A randomised controlled trial published in the nutritional neuroscience journal yielded a positive outcome and improvement in the overall well-being of the patients enrolled.

We explored the dietary habits of acute inpatients. After gathering a detailed diet history, their food habits were compared to a Mediterranean dietary index to assess their dietary patterns. Additionally, variables such as socio-economic characteristics, physical activity, income, BMI, and educational achievement were taken into consideration, with the intention to understand the effect of these factors on a diet consumed by acute patients and the relationship of the diet with their mental wellbeing.

Ultimately, this study aims at an important aspect of preventive mental health, i.e., improved dietary habits (e.g., Mediterranean diet) may contribute to more rapid symptoms resolution and acute stabilization on a short-stay inpatient unit.

## Introduction

Omega-3, omega-6 polyunsaturated fatty acids (PUFA), vitamin D, mineral intake lead to a decrease in the incidence of mental disorders. Decreased levels of folate have been linked to a high risk of depression, dementia, schizophrenia, and lead to psychiatric symptoms in medical patients as well as more severe symptoms in psychiatric patients. Amino acids supplements can be appropriately used for controlling depression, bipolar disorder, schizophrenia, anxiety, attention deficit hyperactivity disorder (ADHD), addiction, and autism. Amino acids get converted to neurotransmitters that help to reduce symptoms of mental disorders [1]. In the last few years, there have been several studies identifying the importance of a healthy diet in mental disorders [2]. One of the studies suggests the Japanese diet rich in vegetables, fruits, mushrooms, soy leads to less depressive symptoms [3]. The western diet is associated with increased anxiety in men and women, and the Norwegian diet decreases depression and anxiety. Two prospective studies suggest that diet quality influences the risk of depressive illness in adults over time with the Mediterranean diet reducing the severity [4] and consumption of processed foods increasing the severity of depression [5]. 

Mood disorders such as depression, bipolar disorders are very common in acute psychiatric units [6]. Various studies have argued that depression is associated with deficiencies in neurotransmitters, such as serotonin, dopamine, noradrenaline, and gamma-aminobutyric acid (GABA) [7-11]. As reported in several studies, the amino acid tryptophan, tyrosine, phenylalanine, and methionine are often helpful in treating many mood disorders, including depression [12-15]. Tryptophan is a precursor of serotonin and converts to serotonin when it is taken alone on an empty stomach. Therefore, tryptophan can induce sleep and tranquillity and in cases of serotonin deficiencies, restoring serotonin levels leads to diminished depression [14].

The incidence of major depression has increased, as the consumption of fish and other sources that are rich in omega-3 fatty acids has decreased. Eicosapentaenoic acid (EPA) is converted into docosahexaenoic acid (DHA), the two omega-3 fatty acids found in fish oil. EPA involves neurotransmitters to elicit antidepressant effects and converts into prostaglandins, leukotrienes, and other chemicals the brain needs [1].

Psychological stress increases the pro-inflammatory cytokines and subsequent inflammation also contributes to depression; suggesting a bidirectional relation between psychological stress and inflammation [16]. Highly reactive oxygen species and high oxidative stress lead to inflammation, which further contributes to depression. Consumption of diets rich in antioxidants, vitamins, minerals, and fibre is associated with reduced systemic inflammation [17]. On the other hand, diets that are low in essential nutrients, such as magnesium and western-type dietary patterns [18] are associated with increased systemic inflammation. 

There is a global shift to a diet rich in refined carbohydrates. Hyperglycemic diets and diets high in glycemic load (GL) lead to systemic inflammation [19]. Refined sugars and saturated fats decrease the expression of neurotrophic factors that predisposes to depressive illness [20]. Thus, modifying inflammatory, oxidative, and neurotrophic factors, quality of diet, influences the course and severity of depressive diseases, which is further needed to be tested. A study that involved the ecological analysis of schizophrenia and diet concluded that increased consumption of refined sugar results in a lack of substantial improvement in patients with schizophrenia, which was measured by the number of days spent in the hospital and low social functioning [21]. A meta-review of randomized control trials suggests that anti-inflammatory nutrients, such as omega-3 and folate-based compounds, may be useful for mental disorders, including bipolar disorder and schizophrenia. Because psychotic disorders are associated with inflammation, these adjunctive compounds may have neuroprotective effects in the early stages of illness among young people [22], improving cognitive outcomes for some patients. 

Mediterranean dietary patterns are comprised of: abundant plant foods (fruits, vegetables, bread, other forms of cereals, pulses, nuts, and seeds); minimally processed, seasonally fresh, and locally grown foods; fresh fruits as the typical daily dessert with sweets elaborated from nuts, olive oil, and concentrated sugars or honey that are consumed during feast days; olive oil as the principal source of dietary lipids; dairy products (mainly cheese and yogurt) consumed in low to moderate amounts; fewer than four eggs consumed per week; red meat consumed in low frequency and amounts; wine consumed in low to moderate amounts, and generally taken with meals. Such a dietary pattern assures a sufficient intake of certain nutrients that have been related in some way with a reduced risk of several chronic diseases. Various scores or indexes have been developed to assess the adherence to the Mediterranean diet pattern in the population and to link such practices with several nutrient-related disorders.

A trial conducted in clinically depressed participants [23], emphasized the importance of the Mediterranean diet in the reduction of depressive symptoms, with a significant number of participants achieving remission compared to the control group. Many other randomized control trials have replicated these findings of the Mediterranean diet reducing symptoms in people with moderate to severe depression [24]. As a meta-analysis of 50 studies has shown, the Mediterranean diet significantly reduces inflammatory markers in other (i.e., non-psychiatric) populations, and it is possible that the benefits in people with depression are linked to the anti-inflammatory effects [25]. 

Changing established dietary behaviours is challenging, and this is attributed to factors such as the prevalence of obesity in the population and the addictive nature of high-fat, high-sugar foods. However, the threshold for neural rewards can be changed in favour of preferring healthy over unhealthy food. A Mediterranean diet is healthy and palatable and can become a sustainable part of a healthy lifestyle [26].

As seen above, the role of diet plays a crucial role in contributing to changes in mental health. This is an aspect less addressed by researchers and further not widely acknowledged by physicians. Thus, this study aims to increase the data pool to answer the question of whether dietary patterns have any effect on mental health, particularly the acute psychiatric inpatients. This study seeks to contribute to the growing school of thought that a holistic approach is required for psychiatric treatment, especially healthier diet control. 

## Materials and methods

The study was conducted in an acute inpatient psychiatric unit of an academic hospital. The study was approved by the institutional review board. One hundred non-consecutive inpatients were approached by the researchers, and after their verbal agreement, the Eating Habits Questionnaire (Dana-Farber Institute) was administered. The questionnaire was employed owing to its concise set of questions addressing major food groups and multiple options for recording the frequency of consumption. The questionnaire also entailed some basic questions regarding the demographic characteristics of the study population. The explanation was offered regarding the purpose of the study, and confidentiality was ensured. 

The Mediterranean Diet Quality Index (KIDMED) questionnaire is used to evaluate the adherence to a Mediterranean diet in adolescents. It consists of 16 items, with four questions denoting a negative connotation to the Mediterranean diet (consumption of fast food, baked goods, sweets, and skipping breakfast) and 12 questions denoting a positive connotation (consumption of oil, fish, fruits, vegetables, cereals, nuts, pulses, pasta or rice, dairy products, and yogurt). Questions denoting negative connotations are scored with −1, while positive connotation questions are scored with +1. According to the KIDMED index, a score of 0-3 reflects poor adherence to the Mediterranean diet, a score of 4-7 describes average adherence, and a score of 8-12 good adherence [27]. However, the score was modified to use in adults, as "healthy" with a score of over 4 and "unhealthy" as 3 and under. 

The study was conducted as a descriptive, cross-sectional, correlational design, to examine the relationship between the KIDMED score index (a measure of healthy dietary habits) and body mass index (BMI), gender, level of education, smoking, income, and exercise. The data was analyzed using Statistical Package for the Social Sciences (SPSS Inc., Chicago, IL).

The Mediterranean diet index KIDMED was used to categorize all patients as having a healthy or unhealthy score which corresponded to their dietary habits. 

## Results

Descriptive analysis using frequency and percentages were used to describe the demographic data of this study. Correlation analysis between the KIDMED score (dependent variable) and biosocial variables, age, gender, level of education, race, income, and physical activity (input variables) was done using chi-square, and Spearman's ranked univariate analysis. Further univariate analysis using analysis of variance (ANOVA) was utilized to evaluate for associations between KIDMED scores and Grouped Mental Health conditions. Our study did not correct for confounders and statistical significance in view of the exploratory nature of the study. We did not correct for multiple tests of statistical significance since our study is an exploratory one.

Sample characteristics

The demographic characteristics of the surveyed population are shown in Table 1.

**Table 1:** Sample characteristics (n=100)

Item	Frequency (N)	Percentage (%)
Age in years		
18-30	25	25%
31-40	24	24%
41-50	16	16%
51-60	22	22%
>60	13	13%
Race		
White	79	79%
Black	13	13%
Asian or Pacific Islander	1	1%
American Indian	2	2%
Other	5	5%
Gender		
Male	55	55%
Female	45	45%
Smoker		
Yes	46	46%
No	54	54%
Body Mass Index		
<18.5	2	2%
18.6- 24.9	36	36%
25-29.9	25	25%
>30	37	37%
Level of Education		
Less than High School	16	16%
High School Diploma	25	25%
Vocation/Trade School	4	4%
College/Associate Degree	39	39%
Bachelor’s Degree	16	16%
Income		
Less than 10K	18	18%
10-29K	37	37%
30-49K	27	27%
50-69K	12	12%
70K and above	6	6%
Exercise		
Do not exercise	36	36%
Exercise once a week	5	5%
Exercise 2-4 times/week	32	32%
Exercise 5-7 times/week	14	14%
Not regular	13	13%

The majority of respondents were between 18 years and 50 years old (65%) with a mean age of 43 years. Fifty-five (55%) percent of the surveyed population were females. Overall, seventy-nine (79%) percent of those surveyed identified as Caucasian.

The mean BMI computed from weights and heights taken at the time of admission was 29.8. Sixty-two percent of those studied were classified as either overweight or obese, with 37% in the latter category. Overall, less than one in two received as much as a high school diploma. Surprisingly, 39% reported having obtained a college or associate college degree. There was an almost equal distribution between smokers and non-smokers, with the latter predominating at 54%. Sixty-four percent (64%) of those studied admitted some level of physical exercise on a weekly basis, with nearly 50% exercising at least once weekly. With reference to household income, 1 in 2 grossed less than $30,000 yearly (55%) as combined household earnings.

**Figure 1:**
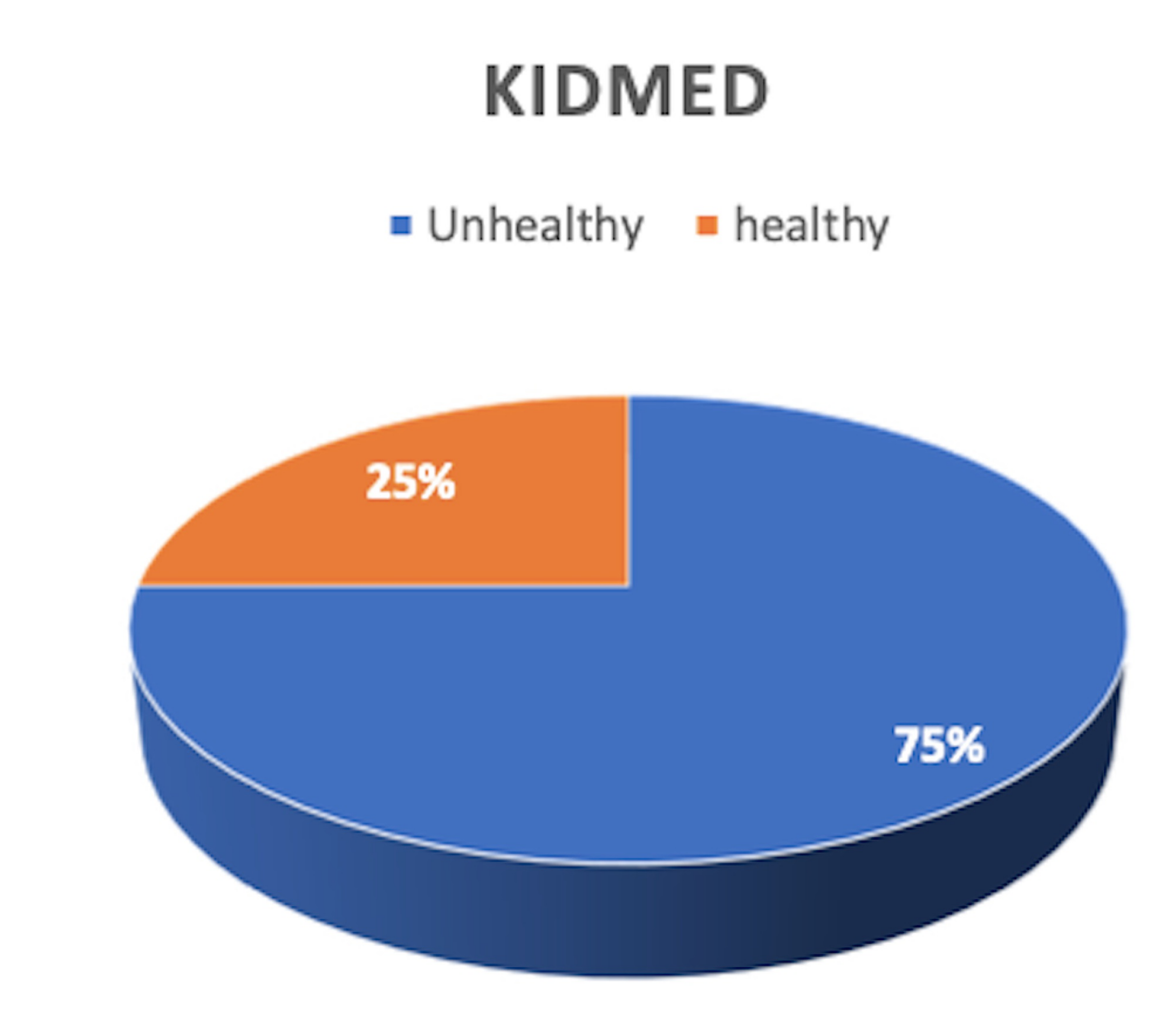
Percentage distribution of study sample according to the KIDMED score KIDMED: Mediterranean Diet Quality Index

Figure 1 shows that the majority of the participants (75%) are engaged in poor eating habits according to the KIDMED score which is (the frequency distribution of the Mediterranean diet). Seventy-five percent of respondents were categorized as having a poor dietary pattern based on the KIDMED score no greater than 3.

Correlations

A positive correlation between education level and the KIDMED score (0.196) was found using Spearman's ranked analysis. Conversely, a negative correlation coefficient (-0.124) was found between BMI and the KIDMED score. Both findings yielded a statistical significance with p values of 0.05 and 0.04, respectively (Table 2).

**Table 2:** Correlation between KIDMED score and age, gender, education, race, BMI, income and physical activity; chi- square analysis * indicates p value <0.05 ** indicates Spearman Coefficient of range >1/-1 KIDMED: Mediterranean Diet Quality Index; BMI: body mass index.

	Variable	S. Coef	P value
	Age	-0.124	0.223
	Gender	0.43	0.407
KIDMED Score	Education	1.98**	0.05*
	Race	0.71	0.48
	Income	0.04	0.97
	BMI	-0.124**	0.04*
	Physical Activity	0.149	0.138

The study failed to show any statistical significance for correlations between age and KIDMED score (p-value = 0.223) using Spearman's coefficient analysis. Similarly, no significance was found between the KIDMED score (dependent variable) and gender, level of education, BMI, income, and physical activity, using chi-square analysis.

**Figure 2:**
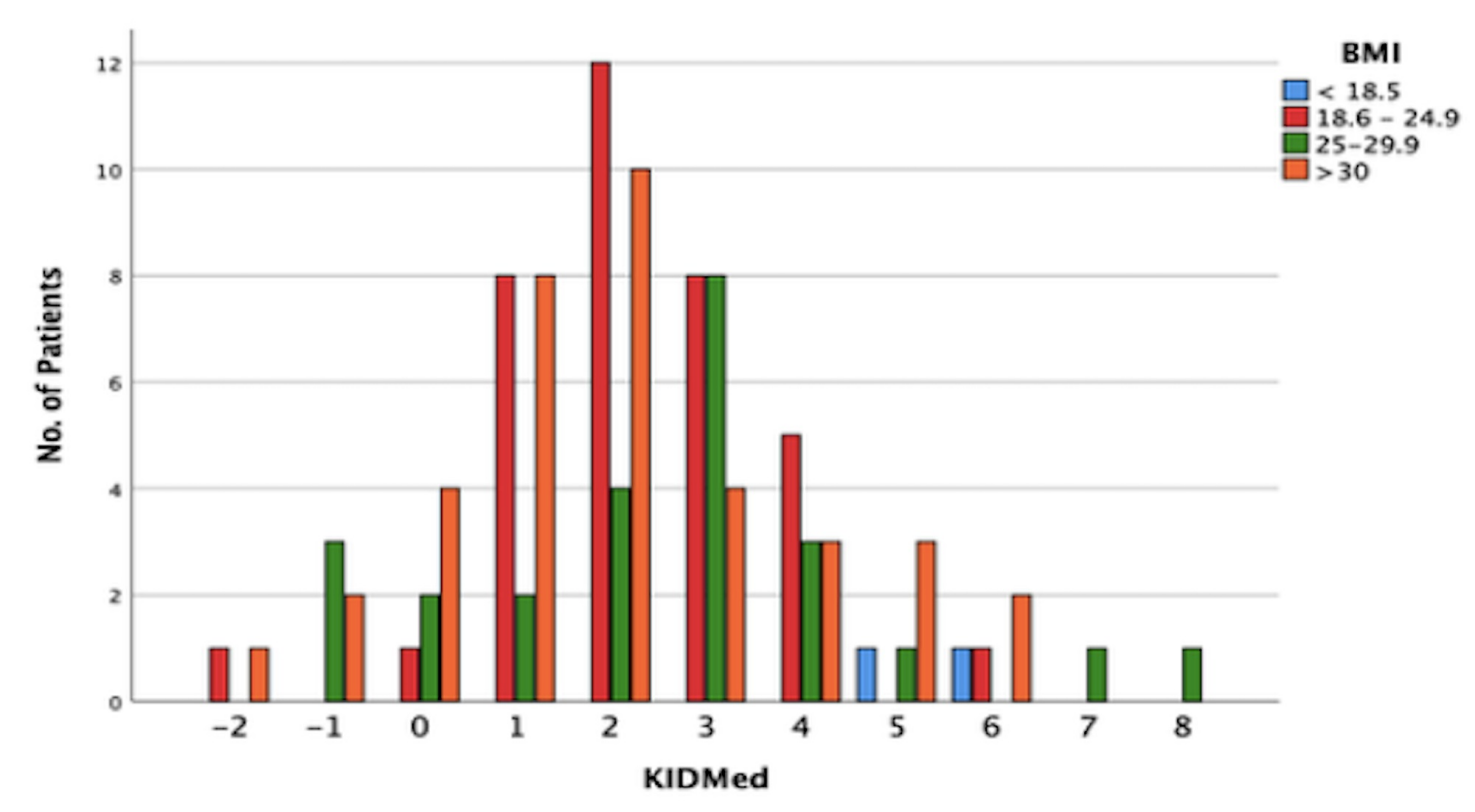
Bar histogram showing BMI groups vs KIDMED score KIDMED: Mediterranean Diet Quality Index; BMI: body mass index.

Figure 2 is a bar chart/histogram of the KIDMED score and BMI.

Grouped mental health disorders and KIDMED scores

Analysis for correlation between Grouped Mental Health conditions and KIDMED score using the univariate ANOVA test failed to show any statistically significant association (p-value =0.463). Table 3 is an illustration of Grouped Mental Health conditions, and the concomitant means the KIDMED score for each group. Overall, the mean KIDMED scores for all the groups were poor and failed to identify any trends with an increasing number of concomitant mental health conditions (multiple diagnoses).

**Table 3:** Overlap of mental Illness in the patient population + mean KIDMED scores >4 KIDMED Classified “Healthy” <4 KIDMED Classified “Unhealthy” KIDMED: Mediterranean Diet Quality Index

Mental Illness	Frequency	Mean KIDMED Score	Std. Deviation
Mood + Anxiety	14	2.07	1.54
Mood + Substance Abuse	16	2.06	1.61
Psychotic + Substance Abuse	6	2.83	2.22
Anxiety + Substance Abuse	6	2.17	1.16
Psychotic + Anxiety + Substance Abuse	4	2.25	0.95
Mood + Psychotic + Substance Abuse	5	2.6	1.67
Mood + Anxiety + Substance Abuse	11	2.82	2.04
Mood + Anxiety + Substance Abuse + Others	4	1.5	2.51

Correlation between sugar consumption and psychosis

Significantly, the study revealed a strong positive correlation between sugar consumption (number of daily teaspoonfuls of added sugar) and psychotic individuals using the chi-square analysis (p-value = 0.001). This result closely identifies with those found in existing literature showing an inverse relationship between sugar intake and severity of psychosis. 

## Discussion

The frequency and the mean scores in this study identified a majority of acute psychiatric inpatients exhibiting unhealthy dietary habits that are known to cause deficiencies in essential nutrients and minerals that can exacerbate mental illnesses. The study further highlights that the causes of mental health conditions are multifactorial, and not all biopsychosocial factors show a strong direct association. Confounders such as the concomitant administration of antipsychotic medications are known to cause metabolic disturbances such as glucose dysregulation, hyperlipidemia, weight gain, and may also impact the role of diet directly [28]. Importantly, the apparent underreporting of alcohol consumption cannot be excluded from the overall accuracy and veracity of data collected. Alcohol consumption decreases serum high-density lipoprotein (HDL) cholesterol and also leads to hypertriglyceridemia, hyperinsulinemia and high waist circumference. In our study, 69 patients were diagnosed as having a substance use/abuse problem, while 70 claimed liquor consumption of less than once weekly. Although 57 admitted that they drank one bottle of beer 1-3 times per month, an accurate determination for other liquor consumption could not be made. 

In a patient population with persistent mental illnesses, the prevalence of a constant low mean KIDMED score (2.9) and covalently, poor eating standards, can be partially attributed to social impairment and diminished quality of self-care as a result of their mental illness. However, data regarding the impact on the quality of life and disability was not recorded.

Evidence exists that substance abuse is rampant [29]; as well as in our population (64% of the population), mean KIDMED scores are 2.9, and the mean BMI is 29.8. In the case of active addiction, malnutrition occurs in 5%-30% of the cases, while in recovery, substance abuse patients report concerns with unhealthy eating patterns, unhealthy weight gain, and development of obesity with weight gains of approximately 3 kilograms over 12 weeks common. 

Seventy percent of the study population were suffering from depression, which is accompanied often by decreased psychomotor state and appetite changes. Thus, further contributing to poor eating habits in our patient population. The diagnosis of depression was made by the licensed psychiatrist, keeping in mind to fulfill 5 out of 9 criteria for depressive disorder. The overlay of prominent mood disorder (70%) and poor eating habits (mean KIDMED of 2.9) can be attributed to the existing evidence of a bidirectional involvement of depression and poor diet. Additionally, there is compelling evidence that regular exercise is protective against depression [30]. Our analysis comparing the mean levels of physical activity in depressive patients are seen as 58% of patients in our sample population with mood disorders "don't exercise or exercise <1 time/week". It highlights the importance of intervention of physical activity in the active treatment of depressive inpatients. 

Unhealthy food and inactivity have been part of the culture of mental health treatment. These practices are seen as usual or as self-comfort strategies by the physicians. Patients are guided by the psychiatrists, sometimes with the help of nutritionists, to not indulge in unhealthy food practices. Ultimately, it depends on the adherence of the patients. Sometimes, mental health practitioners may not feel competent to provide advice on nutrition and diet; the evidence suggests that detailed advice may not be necessary. Recommendations and encouragement to follow national guidelines for dietary and exercise practices should be part of care for all people with mental illness and especially depression.

In our study, in an acute inpatient setting, 75% of the overall population displayed an unhealthy diet based on their KIDMED scores. The results of the chi-square tests comparing biosocial factors vs KIDMED scores revealed a correlation between the level of education, BMI, and the KIDMED index. However, the other biosocial factors (age, gender, income, and physical activity) did not yield significant results (refer to Table 2). Several factors served as limitations in the current study. I) The low sample size did not accommodate the derivation of correlations. II) The lack of a control group contributed towards not being able to draw effective correlations. III) The KIDMED index gives an estimated score ranging from -2 till 8. This scale, though standard for effective implementation of a Mediterranean diet, is limited in the inclusion of the detailed variety of diet types in the Dana-Farber Institute Nutritional Questionnaire. IV) The mean KIDMED score of our sample group is (2.9) and a median of (2.0). This prevents the sample size from being stratified effectively into healthy and unhealthy groups. V) Recall bias exhibited by the patients and overestimating the consumption of foods they understand to have higher nutritional value. VI) Methods for accurately measuring people's dietary intakes remain problematic. Diet quality is most often measured using a priori dietary quality index derived from recommended dietary guidelines or Mediterranean indices. They commonly identify two main dietary patterns that reflect dietary habits; often labelled as (Healthy) and (Unhealthy). An issue with employing a priori diet quality score, such as a Mediterranean-style dietary index is problematic in non-Mediterranean cultures, as in our study. However, newer methods, such as the use of DII are less influenced by cultural contexts and need to be more widely applied. VII) Studies based on diet intake are fraught in general with limitations. Covariance between health behaviours such as diet, physical activity, and smoking are all associated with depression in a bidirectional manner, as well as being correlated with each other. Understanding how each interacts with the other can complicate the interpretation of the results of observational studies. 

Patients with schizophrenia are known to have poor dietary habits. Forty to 62% of people with schizophrenia are obese or overweight. High morbidity and mortality in schizophrenia may be attributed to an unhealthy lifestyle such as poor diet, lack of exercise, smoking, and substance abuse. Increased sugar and processed diet consumption are thought to be characteristic of people with schizophrenia. An ecological study that used data from the World Health Organization (WHO) showed negative outcomes in schizophrenic patients consuming high levels of refined sugar. These assumptions hold true even in our sample population. 55.1% of the patients with psychosis are obese or overweight, with a mean BMI of 27.36. There is a marked increase in sugar consumption on a daily basis in our patients suffering from psychosis with a p-value of 0.01. 

## Conclusions

The findings of this study established the prevalence of unhealthy eating habits in psychiatric inpatients with a significant association between ascending levels of education with improved diet, and of lower BMI and a better diet. Our finding suggests that improved dietary habits (e.g., Mediterranean diet) may contribute to more rapid symptoms resolution and acute stabilization on a short-stay inpatient unit.
